# Evaluation of Interdental Papilla Regeneration Using Injectable Hyaluronic Acid: A Clinical Study

**DOI:** 10.7759/cureus.64510

**Published:** 2024-07-14

**Authors:** Avneet Kaur, Pramod W Waghmare, Vidya Dodwad, Vishakha Patil, Mansi Mukul, Rashida Husain

**Affiliations:** 1 Periodontology, Bharati Vidyapeeth (Deemed to be University) Dental College and Hospital, Pune, IND

**Keywords:** gingival recession, black triangle, hyaluronic acid, minimally invasive reconstruction, deficient papilla

## Abstract

Aim: This study aimed to assess the efficacy of hyaluronic acid (HA) gel injections in deficient papillae and record the effects for four weeks.

Materials and methods: Fifteen deficient class 1 papilla sites according to Nordland and Tarnow classification were included. After scaling and root planing, 0.5 ml HA gel was injected into the papillae. Measurements of black triangle height (BTH) and black triangle area (BTA) from the contact point to the most coronal level of the visible papilla tip were done on the clinical photographs using ImageJ software at baseline, one week, and four weeks postoperatively, and a comparison was made. Descriptive data were examined using the mean and standard deviation (SD). Paired t-test was used for intragroup comparisons, with p-values <0.05 considered significant. All the data analysis was done using SPSS software version 25.0.

Results: There was a mean decrease in the BTA from baseline (0.54 mm^2^), one week (0.13 mm^2^), to four weeks (0.26 mm^2^) with a slight loss of papilla volume from the first week to the fourthweek, and this decrease in area was statistically significant. A mean decrease in the BTH throughout the follow-ups from baseline (1.36 mm) to the first week (0.30 mm) to the fourth week (0.73) was recorded with a slight loss of papilla volume from the first week to the fourthweek, and this decrease in height was also statistically significant (p < 0.05). However, the decrease in the BTA and BTH recorded was more from baseline to the first week as compared to baseline to the fourth week postoperatively.

Conclusion: HA gel is an effective treatment for minimally invasive papilla augmentation, particularly in class I Nordland and Tarnow papilla deficits.

## Introduction

The interdental papilla is a portion of the gingiva that occupies the gingival embrasure, which is the interproximal space below the area of tooth contact. It is keratinized gingival tissue covered by stratified squamous epithelium [1]. It is triangular and pyramidal when viewed two- and three-dimensionally, respectively. The lingual and facial surfaces taper toward the interproximal contact area, while the mesial and distal surfaces are slightly concave. It is made up of dense connective tissue that is covered by the oral epithelium. The shape, position, and presence of interdental papilla are based on the underlying alveolar bone, the proximal contact point, and the area of approximal teeth [2]. Cohen was the first to describe the morphology of interdental papilla.

The absence of any one of the above factors can lead to an empty space between teeth known as the black triangle. Several other factors can also result in black triangles, which include periodontal disease, root angulations, triangular‑shaped crowns, faulty brushing technique, aging, tooth loss, and length of the embrasure area [3,4]. A deficient interdental papilla can cause phonetic problems and food impaction and is aesthetically displeasing. This poses a real challenge for periodontists and restorative dentists to regenerate or reconstruct the lost papilla. Recently, hyaluronic acid (HA) has begun to be used in the reconstruction of interdental papillae. HA is a structural and physiological component of the extracellular matrix, contributing to tissue homeostasis [5]. Its physiologic roles include cell-cell interactions, pain regulation, wound healing, angiogenesis stimulation, and collagen synthesis [6]. It is non-immunogenic, biocompatible, and biodegradable, making it suitable for biomedical applications. Modification of HA molecules through cross-linking can create gels with strong and rigid structures, thus improving mechanical properties, making them suitable for medical applications and further treatment for six to 12 months [7,8].

HA is a linear biopolymer that occurs in human tissues and synovial fluid. Since HA is widely used as an effective filler to improve facial volume, it has also been successfully used in interdental papilla treatment [9]. An experimental study investigating the use of HA in interdental papilla reconstruction was conducted in 2010, and many clinical studies have been conducted since then. When the injection is stopped, the interdental papilla loses the gained volume, which will take six months [10,11].

Some data reporting results after HA injections have recently been published. Becker et al. examined outcomes at 14 clinical sites following up to three injections (three weeks apart) of HA gel 2-3 mm above the tip of the papilla. The study found that HA can effectively treat interdental papilla loss and maintain benefits for up to six months. Another clinical trial also evaluated the ability of HA gel to fill black triangles; most reported having adequate papillas with a six-month follow-up period. However, there are currently not many studies regarding the same. Therefore, this study was conducted to evaluate the regeneration of interdental papilla using injectable HA gel.

## Materials and methods

Out of all the patients visiting the Department of Periodontology, Bharati Vidyapeeth (Deemed to be University) Dental College and Hospital, Pune, subjects willing to participate in the study were shortlisted. Subjects aged 19-50 years old with at least one class I (according to Nordland and Tarnow classification 1998) deficient papillary site were included in the study. Furthermore, subjects having plaque index (Silness and Loe) and gingival index (Loe and Silness) scores between 0 and 1 were finalized for the study.

Subjects with smoking and tobacco chewing habits were excluded from the study. Those having periodontitis or periodontal pocket >4 mm, gingival enlargement, autoimmune diseases, systemic diseases, and conditions (e.g., chronic inflammation, hepatitis, rheumatoid arthritis, atherosclerosis, and diabetes mellitus), allergy to any of the medications or history of food allergy were excluded from the study. Pregnant and lactating mothers were also excluded. Subjects with <2 mm of keratinized gingiva and fixed restorations on study teeth were excluded.

**Figure 1:**
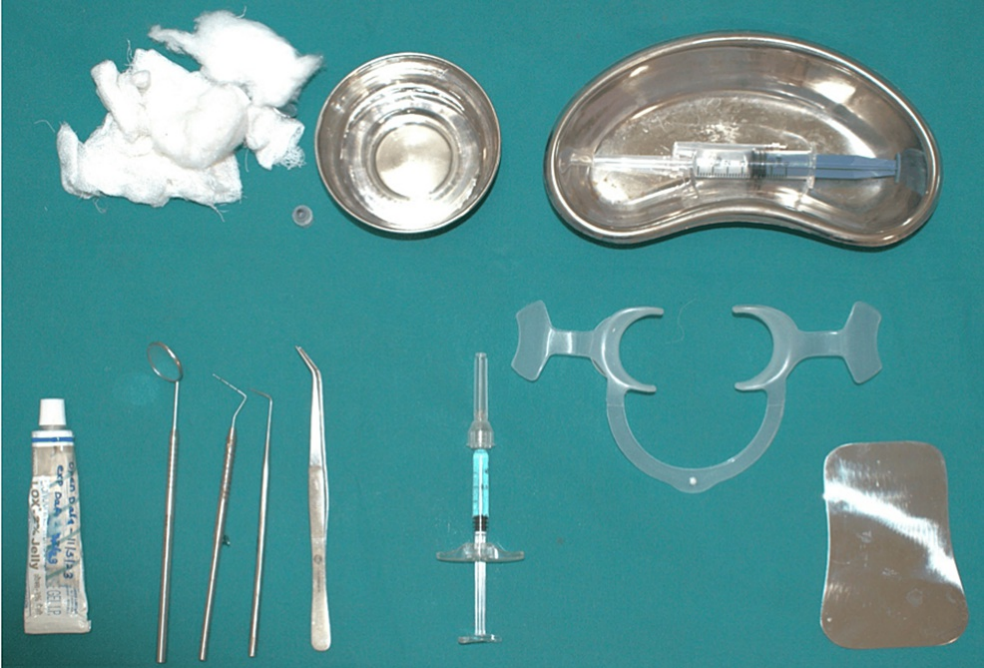
Armamentarium

A detailed case history of all individuals was obtained. A precise oral examination was carried out with proper illumination using a mouth mirror and a University of North Carolina-15 (UNC-15) probe calibrated in millimeters. After taking a detailed case history and clinical examination, the individuals who fulfilled the inclusion and exclusion criteria were included in the study. All participating patients submitted written consent indicating their full agreement to participate in this study (Figure 1).

Procedure

**Figure 2:**
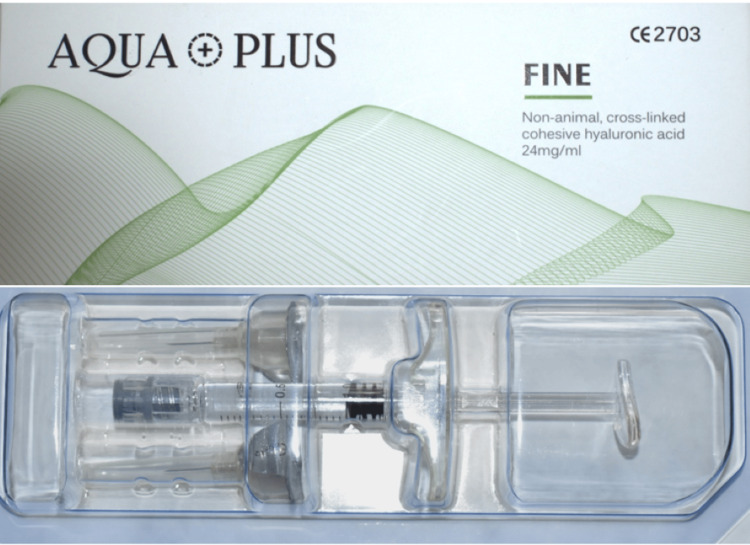
Hyaluronic acid filler injection

A group of 15 subjects was selected. Subjects with Nordland and Tarnow class I were included in the study. The subjects were subjected to thorough scaling, root planing, and polishing. Oral hygiene instructions were given. The subjects were recalled after one week. Black triangle parameters including area and height were recorded. Digital clinical photographs were taken by a professional photographer from the same distance using the same digital camera with the same lens under the same lighting conditions and were assessed for black triangle parameters using digital software. All subjects received HA injections. Local anesthesia was administered 2-3 mm apical to the tip of the papilla, followed by 0.2 mL of HA (Figure 2).

Oral hygiene instructions were given to the subjects. The subjects were instructed to brush twice daily using a modified bass brushing technique with a soft bristle brush. The subjects were recalled after one week followed by four weeks for follow-up. Black triangle parameters were calculated at every interval, and the difference was recorded by a third person to meet the blinding criteria. The difference between the black triangle parameters pre- and post-treatment was evaluated to assess the growth of interdental papilla. A 23-gauge needle was used to inject the gel 2-3 mm apical to the papilla tip, with a coronal orientation. The patient was directed to refrain from brushing for 24 hours before starting routine oral hygiene practices, including avoiding the use of dental floss at treatment sites. The patients were monitored at baseline, the first and fourth weeks after surgery. The photographs were taken under identical lighting conditions, using the same lens and focal length. The patients sat upright and stared straight ahead. The same shooting positions were employed on each occasion. Every location was estimated separately. We measured the distance in millimeters between the contact point (complete interproximal gap fill) and the visible interdental papilla tip's most coronal level. The change in the BTH and BTA between the first and fourth weeks was compared to the baseline.

**Figure 3:**
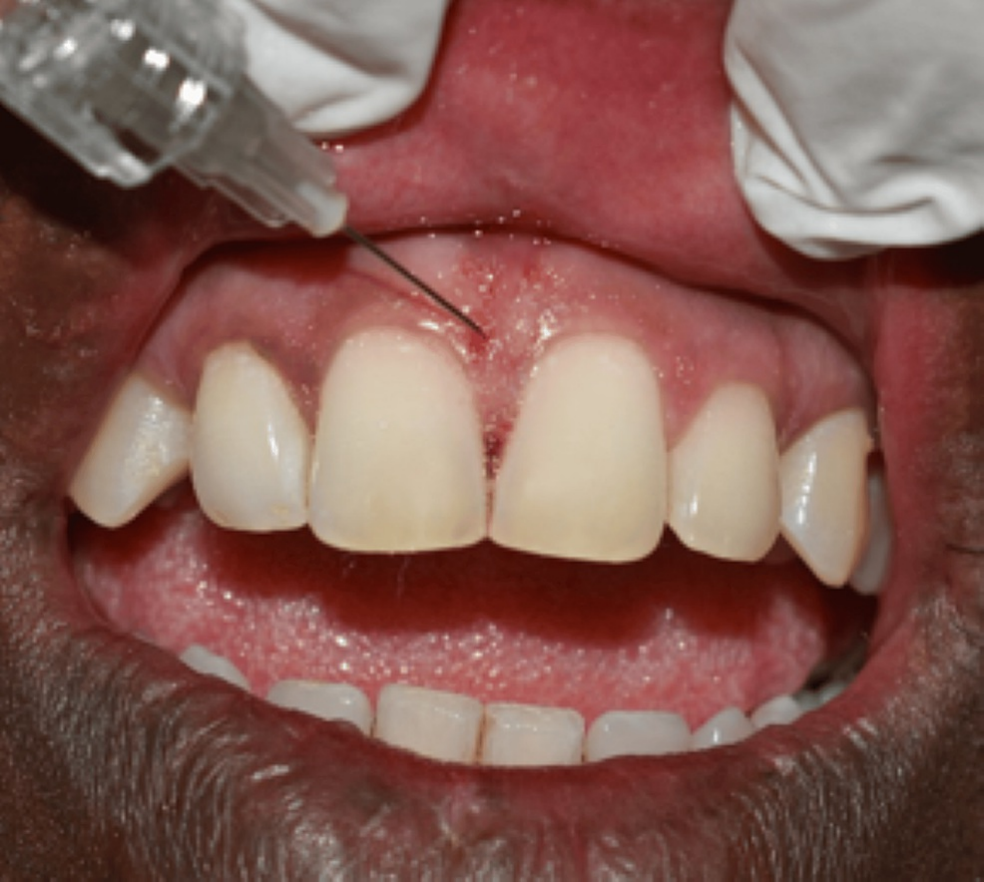
Baseline presentation of the first case Black triangle area (BTA) = 1.495 mm^2^, black triangle height (BTH) = 2.497 mm

**Figure 4:**
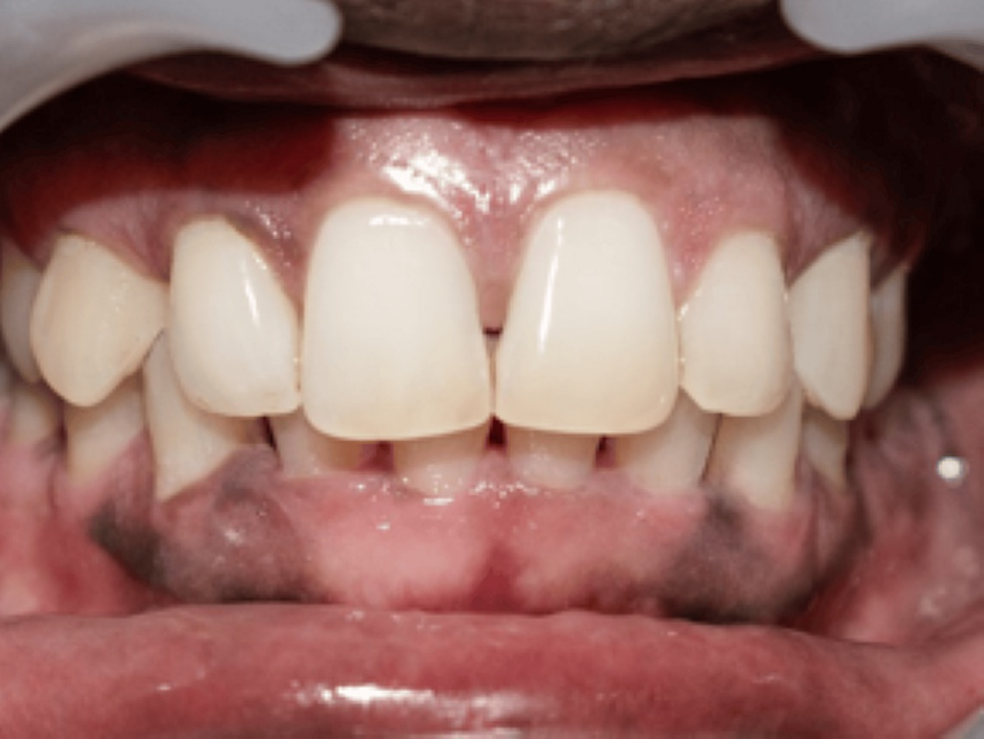
One-week follow-up of the first case Black triangle area (BTA) = 0.34 mm^2^, black triangle height (BTH) = 1.11 mm

**Figure 5:**
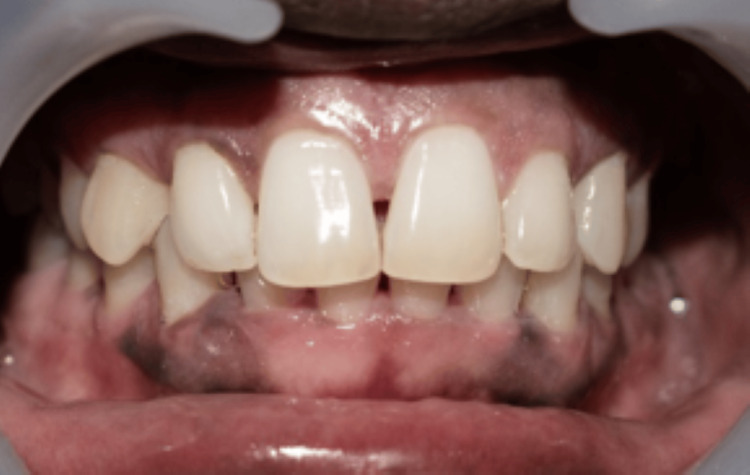
Four-week follow-up of the first case Black triangle area (BTA) = 0.57 mm^2^, black triangle height (BTH) = 1.31 mm

Out of all the 15 cases, 2 cases have been highlighted in the following article. All the cases were followed up on the first week and fourth week. In the first case, the measurements of the BTA and BTH at the baseline were 1. 495 mm^2^ and 2.497 mm. The one-week follow-up showed a decrease in the baseline measurements with the BTA 0.34 mm^2^ and BTH 1.11 mm. There was an increase in the measurements in the fourth week, but it was considerably less than the baseline value, which were BTA of 0.57 mm^2^ and BTH of 1.31 mm (Figures 3, 4, 5).

**Figure 6:**
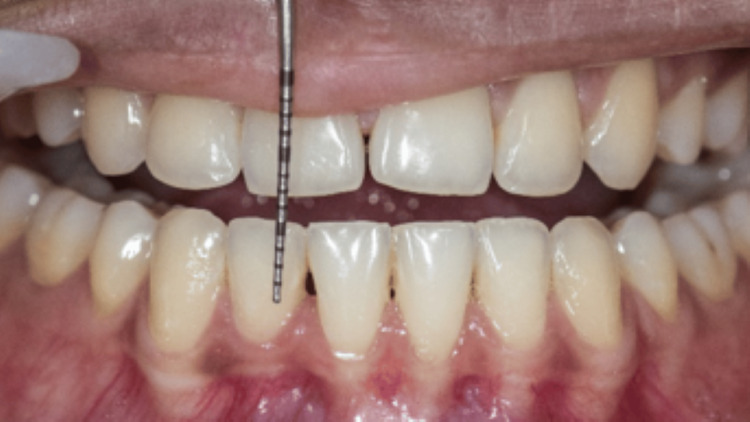
Baseline presentation of the second case Black triangle area (BTA) = 0.71 (41-42),0.32 mm^2^ (31-41), black triangle height (BTH) = 1.87 (41-42), 1.46 mm (31-41)

**Figure 7:**
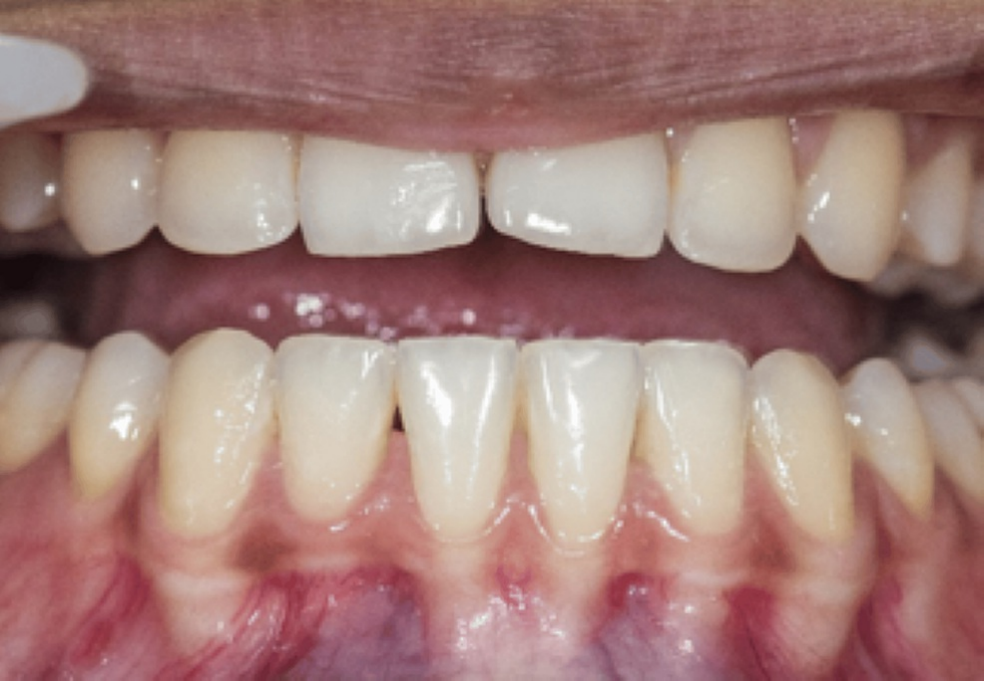
One-week follow-up of the second case showing a decrease in the black triangle area and height between teeth 41-42 and complete fill of the black triangle between teeth 31 and 41. Black triangle area (BTA) = 0.38 mm^2 ^(41-42), black triangle height (BTH) = 1.07 mm (41-42)

**Figure 8:**
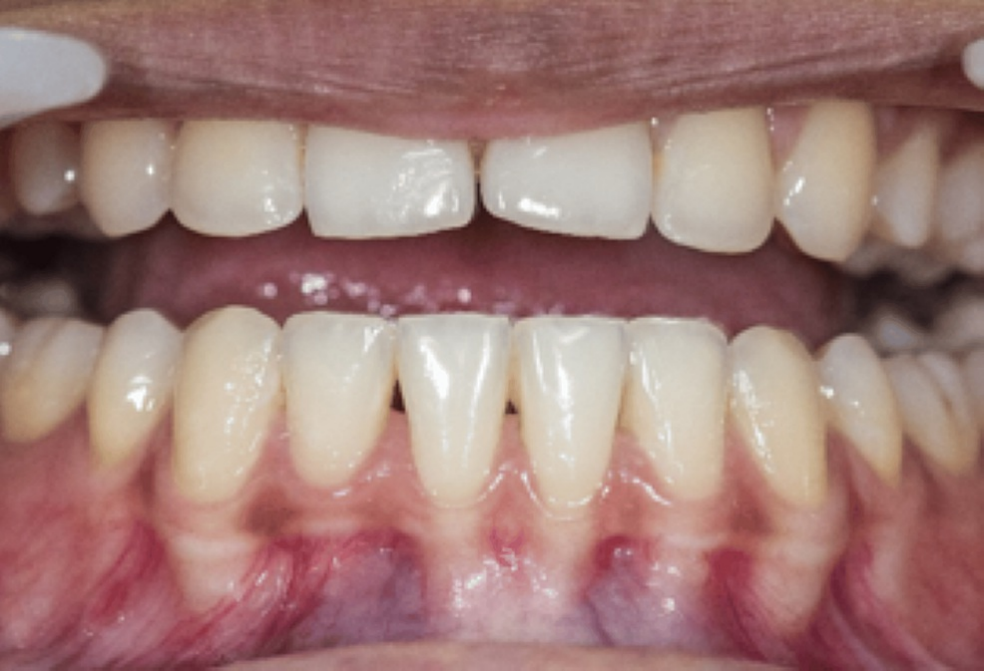
Four-week follow-up of the second case showing an increase in the black triangle area and height between 41 and 42 and between 31 and 41 compared to one week due to the degeneration of hyaluronic acid filler. Black triangle area (BTA) = 0.67 mm^2^ (41-42), 0.26 mm^2^ (31-41), black triangle height (BTH) = 1.58 mm (41-42), 0.66 mm (31-41)

The second case presented with two black triangle sites between teeth 41 and 42 and teeth 41 and 31 according to the Federation Dentaire Internationale (FDI) notation. The measurements of the BTA at the baseline between teeth 41 and 42 were 0.71 mm^2^, whereas the BTH was 1.87 mm. The measurements of the BTA at the baseline between teeth 41 and 31 were 0.32 mm^2^, whereas the BTH was 1.46 mm. The one-week follow-up showed a complete black triangle coverage with the fillers between teeth 41 and 31, whereas a decrease in the baseline measurements was observed at the site between teeth 41 and 42 with a BTA of 0.38 mm^2^ and BTH of 1.07 mm. The black triangle was again noted in the region between teeth 41 and 31 at the end of four weeks with a BTA of 0.26 mm^2^ and BTH of 0.66 mm. Although there was an increase in the measurements of the site between teeth 41 and 42 at the fourth-week follow-up, they were considerably less than that at the baseline with a BTA of 0.67 mm^2^ and BTH of 1.58 mm (Figures 6, 7, 8).

Data were entered in Microsoft Excel (Microsoft Corporation, USA). Data normality was explored using Kolmogorov-Smirnov and Shapiro-Wilk test, suggesting p > 0.05. Descriptive data were examined using mean and standard deviation (SD). Paired t-test was used for intragroup comparisons, with p-values <0.05 considered significant. All data analysis was performed with IBM SPSS Statistics for Windows, version 25.0 (released 2017, IBM Corp., Armonk, NY).

## Results

**Figure 9:**
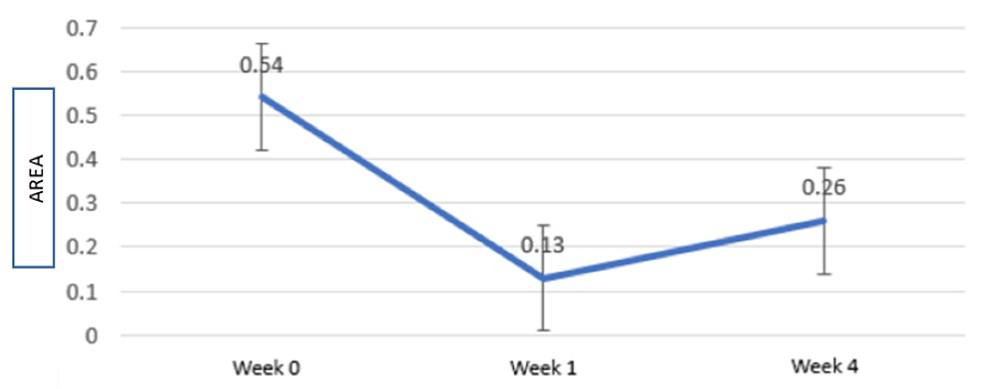
Intragroup comparison for the mean change in the black triangle area with error bars x-axis: time, y-axis: black triangle area (mm^2^)

The t-test paired comparisons revealed a significant increase in papilla size between the baseline and first week compared to the baseline and fourth week. Table 1 and Figure 9 show the intragroup comparison for the BTA (Figure 9, Table 1).

**Table 1:** Intragroup comparison for the black triangle area (BTA) A p-value of <0.05 is considered significant.

Subjects(Black Triangle Area)	Week 0 (baseline mm^2^)	Week 1 (mm^2^)	Week 4 (mm^2^)
1	0.48	0	0.132
2	0.95	0	0.22
3	0.165	0	0.51
4	0.079	0	0.027
5	0.03	0	0.019
6	0.254	0	0.108
7	0.586	1.061	0.415
8	0.156	0	0.032^*^
9	0.45	0	0.052
10	0.05	0	0.028
11	0.32	0	0.148
12	1.495	0.34	0.57
13	2.243	0.57	1.053
14	0.82	0.12	0.71
15	0.033	0	0.017
Mean	0.54 (mm^2^)	0.13 (mm^2^)	0.26 (mm^2^)
Standard deviation	0.60	0.29	0.30
P Value	0.05	0.04*	0.03*

**Figure 10:**
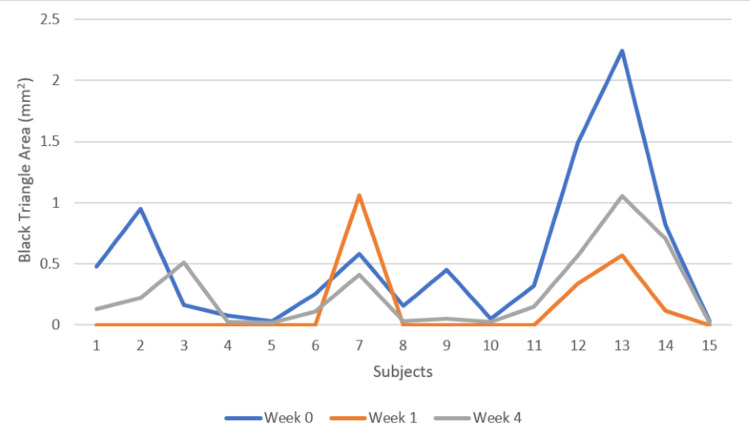
Black triangle area at week 0 (baseline), week 1, and week 4 X-axis: subjects/patients, Y-axis: black triangle area at week 0, week 1, and week 4 (mm^2^)

There is a mean decrease in the BTA from the baseline (0.54 mm), one week (0.13 mm), to four weeks (0.26 mm), and this increase in the BTA was statistically significant (p < 0.05). However, an increase in the BTA was recorded from the first to fourth week (Figures 9, 10).

**Figure 11:**
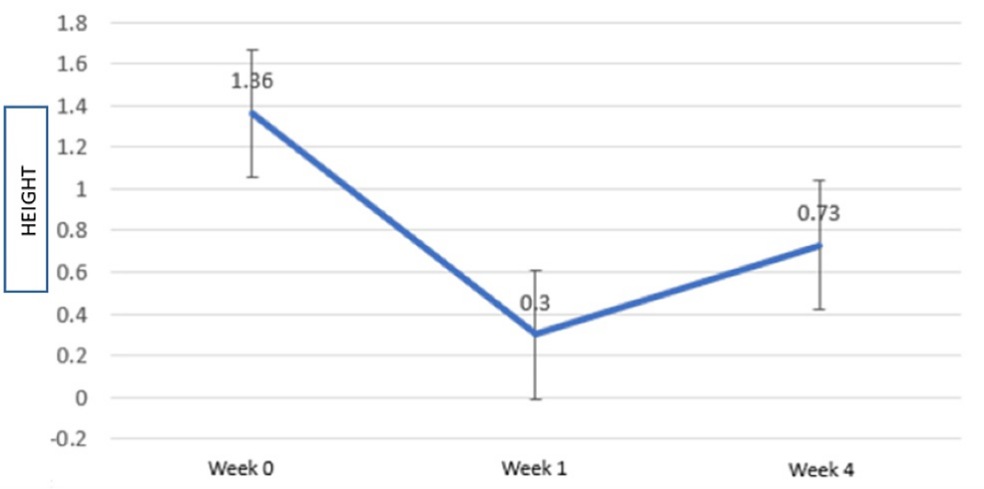
Intragroup comparison for the mean change in the black triangle height with error bars X-axis: time (weeks), Y-axis: black triangle height (mm)

Table 2 and Figure 11 show the intragroup comparison for the BTH (Table 2, Figure 11).

**Table 2:** Intragroup comparison for the black triangle height A p-value of <0.05 is considered significant.

Subjects (black triangle height)	Week 0 (baseline mm)	Week 1 (mm)	Week 4 (mm)
1	1.575	0	0.812
2	1.686	0	0.628
3	0.561	0	0.324
4	0.458	0	0.192
5	0.345	0	0.305
6	1.208	0	0.647
7	2.159	1.885	1.095
8	0.585	0	0.163
9	1.145	0	0.258
10	0.259	0	0.211
11	1.44	0	0.89
12	2.497	1.11	1.31
13	3.881	1.196	2.106
14	2.36	0.35	1.8
15	0.299	0	0.222
Mean	1.36 (mm)	0.30 (mm)	0.73 (mm)
Standard deviation	0.99	0.57	0.59
P-value	-	0.04*	0.03*

**Figure 12:**
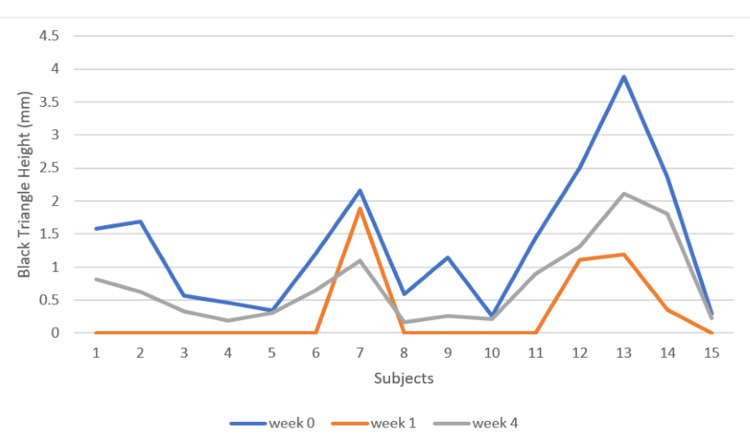
Black triangle height at week 0 (baseline), week 1, and week 4 X-axis: subjects/patients, Y-axis: black triangle height at week 0, week 1, and week 4 (mm)

There is a mean increase in the BTH throughout from the baseline (1.36 mm) to the first week (0.30 mm) to the fourth week (0.73), and this increase in BTH was statistically significant (p < 0.05). However, a decrease in BTH was recorded from the first to the fourth week (Figures 11, 12).

## Discussion

The current clinical study aimed to determine the efficacy of HA gel injection in deficient papillae as a minimally invasive therapy technique for reducing visible black triangles in the anterior aesthetic zone and boosting "pink" aesthetics. The findings of this investigation are encouraging. One week following the HA gel injection, the majority of the treated areas exhibited more papillae and less visible black triangles. Tissue volume decreased over a four-week follow-up period. Furthermore, patients said that the procedure was well tolerated and did not cause pain.

However, the outcomes were not long-lasting, which could be attributed to the type of HA filler utilized and the fact that the injection dose was not repeated. Our findings are broadly consistent with prior research by Awartani et al. [12], Mansouri et al. [13], and Abdelraouf et al. [14], which only showed mean percentage reductions in the black triangle surface area. In our study, the high proportion of papilla fill during the first week was primarily due to the low degree of papilla loss, as only class I deficient papillae were included. Because papillae can be lost for a variety of reasons, they should be cleaned and, if feasible, removed before to HA injection.

Obviously, HA gel cannot heal and replace massive deficits, as demonstrated above. Dentists should evaluate such clinical situations while considering minimally invasive augmentation techniques. Patients should be provided an accurate estimate of the expected outcome of their treatment, as well as information about the possibility of re-injecting HA into the faulty papillae. Many tissues, including gingival connective tissues, contain HA, which is a significant component of the extracellular matrix [15]. This promotes and increases gingival fibroblast cell proliferation, organization, and migration [16]. This was discovered in two clinical comparison investigations that comprised not only a clinical evaluation but also an in vitro analysis of human gingival fibroblast (HGF) proliferation and migration [17].

Ni et al. [17] discovered that HA increased HGF proliferation when compared to the saline solution; however, clinical assessment data from BTA revealed no difference between HA and saline solution after 12 months. The scientists observed an unexpected improvement in the saline solution after a year and attributed it to the gingival papilla's natural creeping and soft tissue renewal, which is generated by local tissue pressure on gingival fibroblasts, which increases cell proliferation [17]. However, Kapoor and Bhardwaj found that only 13.3% of papillary defects treated with the saline solution showed partial healing, leaving the vast majority of papillary abnormalities unaffected [18].

Another study that directly compared the results to saline solution discovered that the surface area of the BTA for HA gel altered considerably (45%) compared to the saline solution (2%), which supports this. However, only a few studies included a direct comparison with a control group, which should be viewed as a limitation for effective comparisons between HA and placebo saline solutions. As a result, more thorough randomized controlled trials are required to determine the impact on papilla healing. The majority of the studies used commercially available crosslinked HA gels.

This might be caused by the fact that HA chains that are not crosslinked into a gel are swiftly degraded by enzymes, such as hyaluronidase and free radicals, implying that free HA or non-crosslinked HA is only available in the skin for a limited time [19,20]. Singh and Vandana [21] described the usage of HA powder at varying doses to avoid side effects. According to the findings, a 5% concentration is superior to 2% and 1% HA since it requires less recuperation. Nonetheless, considering that the majority of the included studies focused on commercially available HA gels; this is the only one that looked into employing HA powder to create a solution.

As a result, it is critical to use caution when interpreting these findings. Many studies used 45-degree-angled needles (23-G, 27-G, 30-G, and 31-G) to inject HA 2 to 3 mm from the papillary tip. Bertl et al. observed two unfavorable reactions to HA injection and advised against developing a reservoir in the mucosa above the mucogingival junction to limit the amount of HA injected (to <0.1 ml instead of 0.18 ml) [22]. Except for discomfort and edema, the majority of trials revealed no side effects. Two studies [23,24] demonstrated a high percentage of papillary healing following five HA injections. However, the authors discovered that increasing the quantity of HA injections did not adequately treat severe papillary abnormalities [25]. As a result, non-invasive treatments should be considered for instances with fewer tissues. Alhabashneh et al. (1989) discovered that maxillary papillary reconstruction was more effective than mandibular reconstruction. According to the researchers, this difference is attributable to symmetric teeth and more keratinized gingiva in the maxillary arch [26].

In terms of gingival phenotypes, the group with a thick gingival biotype had a recurrence and an increase in the area of the black triangle after 12 months [27]. This distinction could be explained by the fact that more gingival tissue thickens the connective tissue, increasing the presence of gingival fibroblasts and collagen fibers [28]. However, the small number of studies that have examined these variables limits our ability to draw definitive conclusions. Thus, more research into the impact of these potential variables is needed. To evaluate papillary reconstructions, all of the studies utilized digital pictures obtained through digital scanning or clinical photography.

For clinical photography, it is crucial to emphasize the consistency of shooting angles for repeated measurements at different time intervals [29], since changes in horizontal and vertical angles might cause clinical images with black triangle distortion. In other studies, baseline images were taken perpendicular to the teeth of interest, with the camera and patient aligned so that Frankfurt's horizontal plane was parallel to the ground. As a result, a photographic device was created to increase the reproducibility of clinical photographs captured in the interdental papilla region, guaranteeing that the shot composition was consistent across all patients. All studies discovered an increase in overall satisfaction following HA injection, which is typically associated to aesthetic appearance [14]. However, any discomfort could be due to using a 23-G needle rather than a thinner one (such as a 30-G needle), which is suggested for these procedures.

Despite the positive findings of the HA injection, various limitations must be addressed in this investigation. One is about HA quality, a single injection dose, the several metrics used to evaluate results, the study's limited follow-up, the unstandardized inclination of teeth, and X-rays not taken. The age range was quite wide for the selected sample. As a result, the findings should be regarded with caution, and more painstakingly planned, controlled research is needed to study and standardize these numerous variables, particularly those associated with implant-supported restorations.

## Conclusions

Within the scope of this study, treating interdental papillary deficiency using a commercially available HA gel appears to be effective and promising. Patients must also be provided a realistic prognosis for the expected results of treatment, as well as an explanation of the option of reinjecting HA into faulty papillae in the long run. The indications must be adequately established and the patients carefully selected. Long-term, randomized clinical studies are required to collect research with a higher level of proof in order to confirm this approach and its various applications. Furthermore, in future studies, the following characteristics must be considered: sample size for greater power and representativeness, product concentration, number of injections, and injection technique. This will make it easy to develop a uniform approach while addressing our patients' aesthetic requirements.
